# A novel zebrafish model to emulate lung injury by folate deficiency-induced swim bladder defectiveness and protease/antiprotease expression imbalance

**DOI:** 10.1038/s41598-019-49152-7

**Published:** 2019-09-02

**Authors:** Gang-Hui Lee, Nai-Wei Cheng, Hsin-Hsuan Yu, Jen-Ning Tsai, Tsunglin Liu, Zhi-Hong Wen, Bing-Hung Chen, Tzu-Fun Fu

**Affiliations:** 1 Department of Medical Laboratory Science and Biotechnology, National Cheng Kung University, Tainan, Taiwan; 2 Institute of Basic Medical Science, National Cheng Kung University, Tainan, Taiwan; 3 Department of Medical Laboratory and Biotechnology, Chung Shan Medical University, Taichung, Taiwan; 4 Clinical Laboratory, Chung Shan Medical University Hospital, Chung Shan Medical University, Taichung, Taiwan; 5 Department of Biotechnology and Bioindusry Sciences, National Cheng Kung University, Tainan, Taiwan; 6 Department of Marine Biotechnology and Resources, Asia-Pacific Ocean Research Center, National Sun Yat-sen University, Kaohsiung, Taiwan; 7 Department of Biotechnology, Kaohsiung Medical University, Kaohsiung, Taiwan; 8 Department of Medical Research, Kaohsiung Medical University Hospital, Kaohsiung Medical University, Kaohsiung, Taiwan; 9 Centers for Biomarkers and Biotech Drugs, Kaohsiung Medical University, Kaohsiung, Taiwan; 10 The Institute of Biomedical Sciences, National Sun Yat-sen University, Kaohsiung, Taiwan

**Keywords:** Transgenic organisms, Zebrafish, Experimental models of disease, Chronic obstructive pulmonary disease

## Abstract

Lung injury is one of the pathological hallmarks of most respiratory tract diseases including asthma, acute respiratory distress syndrome (ARDS) and chronic obstructive pulmonary disease (COPD). It involves progressive pulmonary tissue damages which are usually irreversible and incurable. Therefore, strategies to facilitate drug development against lung injury are needed. Here, we characterized the zebrafish folate-deficiency (FD) transgenic line that lacks a fully-developed swim bladder. Whole-mount *in-situ* hybridization revealed comparable distribution patterns of swim bladder tissue markers between wild-type and FD larvae, suggesting a proper development of swim bladder in early embryonic stages. Unexpectedly, neutrophils infiltration was not observed in the defective swim bladder. Microarray analysis revealed a significant increase and decrease of the transcripts for cathepsin L and a cystatin B (CSTB)-like (zCSTB-like) proteins, respectively, in FD larvae. The distribution of cathepsin L and the zCSTB-like transcripts was spatio-temporally specific in developing wild-type embryos and, in appropriate measure, correlated with their potential roles in maintaining swim bladder integrity. Supplementing with 5-formyltetrahydrofolate successfully prevented the swim bladder anomaly and the imbalanced expression of cathepsin L and the zCSTB-like protein induced by folate deficiency. Injecting the purified recombinant zebrafish zCSTB-like protein alleviated FD-induced swim bladder anomaly. We concluded that the imbalanced expression of cathepsin L and the zCSTB-like protein contributed to the swim bladder malformation induced by FD and suggested the potential application of this transgenic line to model the lung injury and ECM remodeling associated with protease/protease inhibitor imbalance.

## Introduction

Lung injury is one of the pathological hallmarks of most respiratory tract diseases including asthma, acute respiratory distress syndrome (ARDS) and chronic obstructive pulmonary disease (COPD). It involves progressive pulmonary tissue damages which are usually irreversible and incurable. Patients usually receive drug or supportive therapies that relieve symptoms but do not halt or reverse lung damage^1^. Therefore, there is a need to understand the pathophysiological mechanisms so as to develop new preventive/therapeutic strategies and the tools that could expedite drug discovery.

Although the underlying molecular mechanism still remains not completely understood, the progressive lung tissue damage is likely, at least in part, due to the consequence of extracellular matrix (ECM) destruction owing to increased pulmonary proteases activity, which often results from an imbalanced expression of tissue-degrading enzyme and their inhibitors^2–5^. ECM is a supporting structure for morphogenesis and central to lung physiology for maintaining mechanical strength and elasticity, normal interstitial fluid dynamics, effective gas exchange, proper control of cell behavior and tissue repair and remodeling^6,7^. The composition and functions of ECM are regulated by the mutual effect on ECM protein synthesis and the proteolytic activity attributed to the abundant ECM proteases, such as matrix metalloproteinases and cathepsins. Elevated protease activity, either due to increased protease expression, decreased inhibitors or both, is often associated with excessive extracellular turnover and defective bronchopulmonary development and worsening of respiratory diseases^8^. Therefore, strategies and drugs capable of maintaining the balance between prominent proteases and their corresponding inhibitors could lead to the development of an effective treatment^9–11^.

Studies have shown that folate status influences lung development and disease risk, albeit the effects are controversial and the underlying mechanisms remain largely unexplored. Folate, also known as vitamin B9, provides the one-carbon unit for the biosynthesis of numerous macromolecules, including amino acids, proteins, nucleotides, several neurotransmitters and S-adenosylmethionine. S-adenosylmethionine is the primary methyl-donor for DNA, histone, protein and lipid methylation and hence is capable of modulating gene expression in a tissue-, site-, gene- and even gender-specific manner^12–14^. Reduced folate also possesses strong anti-oxidative activity comparable to vitamin C and vitamin E, which is crucial to maintaining proper intracellular oxidative stress and embryogenesis^15–17^. Owing to its multi-activities, folate has been considered as a modifiable environmental factor and a nutraceutical that can modulate the “developmental origin of health and disease (DOHaD)”^18^. Nevertheless, folate metabolic disturbance and folate intake imbalance, either excessive or insufficient, can lead to harmful consequences. High serum folate levels were associated with a low risk of high total serum IgE concentrations, atopy, and wheezing^19^. Nevertheless, folic acid supplementation during pregnancy was reported to be associated with an increased risk of infant bronchiolitis and childhood asthma^20,21^. These conflicting results raise the concern and debate on the policy of mandatory fortification of food and maternal folic acid supplementation, a highly prevalent maneuver world-wide. To date, studies pertaining to the pathophysiological effects between folate supplementation and lung injury in general still remain scarce. Therefore, it is imperative to elucidate how folate affects lung physiology and the pathogenesis of pulmonary diseases to help further discern the nutritional importance of folate.

With the combined advantages of *in vivo* complexity and *in vitro* convenience of high-throughput screening, zebrafish is a vertebrate model prominent for research on various fields including molecular function, diseases mechanism, developmental biology, toxicology and drug discovery^22–27^. Currently, efforts to delineate the pathomechanism of and treatment for diseases involving pulmonary tissue damage have been greatly assisted by rodent models^28–30^. Possessing the advantages of “ *in vitro* convenience” and “ *in vivo* complexity”, zebrafish is ideal to complement rodent for a real-time, dynamic and high-throughput observation and is especially powerful for drug discovery. Specifically to lung research, zebrafish swim bladder provides an ECM environment, which cannot be achieved by cultured cells, and has been suggested to be a simple *in vivo* platform for the study on lung elastin injury and repair^31,32^. Zebrafish swim bladder is a buoyancy organ located in the dorsal-anterior part of the body cavity. Similar to mammalian lung, teleost swim bladder originates from an outgrowth of the foregut endoderm into an out-pocketing gas-filled sac during embryonic development^33^. Zebrafish swim bladder is mainly composed of three distinct tissue layers: endodermal epithelium, the middle mesenchymal layer and the outer mesothelium layer^34^. The gene products closely related to the structural and functional elements of lung tissue are also enriched in swim bladder^35^. Conserved expression of genes involved in Hedgehog (Hh) signaling was observed in developing swim bladder, as in early lung development^34^. Transcriptomic analysis suggested a role for ECM in the development and function of zebrafish swim bladder, as for the human bronchopulmonary system^8,35^. Examination on the zebrafish swim bladder wall revealed a simple structure resembling an inner layer of elastin fibers^31^. The resemblance of structural, ontogenic and molecular characteristics between zebrafish swim bladder and mammalian lung supports the use of the zebrafish swim bladder to model lung physiology and pathology.

Previously, we had established a fluorescent zebrafish transgenic line that displays folate deficiency (FD) upon induction in a timing-, duration- and extent-controllable manner^17^. We found that most of these FD larvae lacked successfully inflated swim bladder. In this study, we examined the histopathological characteristics of these FD larvae and the potential pathways that contributed to the impeded swim bladder formation. Additionally, we identified a previously uncharacterized zebrafish protein, named zebrafish cystatin B (zCSTB)-like, which is structurally and functionally comparable to its human cystatin B ortholog and whose expression was down-regulated in response to FD induction in our zebrafish model. The causal link between FD and the pathological phenotypes was confirmed by folate supplementation. The mechanisms underlying the FD-induced swim bladder malformation and the potential of using this transgenic line to model lung injury are also proposed and discussed.

## Materials and Methods

### Materials

The SMART^TM^ RACE amplification kit was purchased from Clontech. Takara Bio Co. (Mountain View, CA). Restriction enzymes were purchased from New England BioLabs, Inc (Maryland, US). 5-formyltetrahydrofolate was a generous gift from Dr. Moser (Merck Eprova AG, Switzerland). The AB strain zebrafish was purchased from Zebrafish Core Facility at ZeTH/Taiwan. The clones for preparing the WISH riboprobes specific for swim bladder were generous gifts from Dr. ZhiYuan Gong/National University of Singapore. All other chemicals, including elastin stain kit (HT25A-1KT), coenzymes, buffers, amino acids and antibiotics were purchased from Sigma-Aldrich Chemical Co. (Montana, US). The *E. coli* strains and vectors used for cloning and protein expression have been described previously^36^.

### Fish maintenance

Zebrafish (*Danio rerio*, AB strain) were maintained following the standard procedure^37^. The animal studies and all procedures were approved by Affidavit of Approval of Animal Use Protocol of National Cheng-Kung University (IACUC Approval NO. 96062). All procedures complied with the “Guidelines for the Use of Zebrafish” (Chapter 22) in the “A Guidebook for the Care and Use of Laboratory Animals” (3rd edition), published by Taiwan Society of Laboratory Animal Sciences, Council of Agriculture, Executive Yuan, Taiwan (https://animal.coa.gov.tw/html/index_04_3_1.html). The maintenance of FD transgenic lines, Tg(*mpx*:EGFP), Tg(*lfabp*:mCherry/*hsp*:EGFP-γGH) and Tg(*hsp*:EGFP-*γ*GH), and the protocols used to induce folate deficiency were as previously described^17^.

### Whole mount *in-situ* hybridization (WISH)

WISH was performed following the standard protocol^37^ with the probes prepared from the plasmids for Hb9, Acta2, Anax5^38^, zebrafish cystatin B-like protein (zCSTB-like) and cathepsin L. Both zebrafish cathepsin L and CSTB-like cDNA encompassing partial 3′-coding sequence and 3′-UTR were cloned using the following primers: cathepsin L:5′-GGCATATGGCTCCATCTATAGAC-3′(forward) and 5′-CGCTCGAGCATTAGGGGATAG-3′(reverse); zCSTB-like:5′-CGAGAATTCATCAACAATGTCAGAG-3(forward) and 5′-CACGGATCCTGCAAATGCTC-3′(reverse). Digoxigenin-UTP-labeled antisense RNA probes were synthesized using DIG-RNA Labeling kit (SP6/T7) (Roche).

### Histochemical staining

Cryosectioning and H&E staining of zebrafish tissue sections were performed following the protocols in Zebrafish Book^37^. Histochemical staining for swim bladder was performed using elastin/collagen staining kit following the manufacture’s instruction (Sigma-Aldrich).

### Oil red O (ORO) staining and Sudan black staining

ORO staining was performed according to published protocol by Schlegel and Stainier^39^. Generally, zebrafish larvae were fixed by 4% PFA/PBS and soaked in 1, 2-propanediol. Then, larvae were stained with filtered 0.5% ORO in 1, 2-propanediol for 16 hours. ORO-stained larvae were stored in 80% glycerol/PBS after briefly washing in PBS. Sudan black staining were performed following the protocols previously described^40^.

### Cathepsin L and zCSTB-like expression

Reverse-transcription PCR (RT-PCR) was performed using the following primers: cathepsin L: 5′-GCTGATGTTGCTGGGAATAG-3′(forward) and 5′-CCACTATCATAGCCCTAACAGC-3′(reverse); zCSTB-like: 5′-GTGTTTATTGCTGGAGATGAGTGTGC-3′(forward) and 5′-GAGAGTTTAATGTTGAGGAATGAAGCAG-3′(reverse) as previously described. For protein analysis, Western blotting was performed using the polyclonal antibodies against cathepsin L and tubulin (GeneTax, Taiwan)^17^.

### Cloning for zebrafish CSTB-like

The primers used to PCR clone the zCSTB-like coding sequence from zebrafish 5′-RACE-Ready cDNA libraries were designed based on the nucleotide sequence of the uncharacterized protein (GenBank^TM^ accession number EH546362), whose transcript showed an 83-fold decrease in FD larvae. The primer sequences were: 5′-CATATGTCAGAGGCAAAGGCAGG-3′ (forward) and 5′-CGCTCGAGTTTCTTAGGAAGATTCAG-3′ (reverse) with introduced NdeI and XhoI restriction enzyme sites (underlined). The PCR fragments of 318 base-pairs were cloned into the pET43.1a expression vector between its NdeI and XhoI sites, generating zebrafish CSTB-like-His/pET43.1a. Successful cloning was confirmed by restriction enzyme digestion and DNA sequencing. The resulting constructs were transformed into Rosetta (DE3) *E. coli* for protein expression.

### Expression and purification of recombinant zebrafish CSTB-like

The expression and purification of recombinant zebrafish CSTB-like protein was performed using nickel-affinity column following the protocols described previously with minor modifications^41^. In brief, *E. coli* containing zebrafish CSTB-like-His/pET43.1a, that expressed recombinant zebrafish CSTB-like protein with C-terminal His-tag, was grown in enriched Luria broth (2-YT) and induced with IPTG at log phase. After 4 hrs induction, bacteria were pelleted and disrupted in lysis buffer (20 mM sodium phosphate, pH 7.4, 0.5 M NaCl, 5 mM 2-mercaptoethanol, 1 mM MgCl_2_ and 10% glycerol) with sonication. After removing the majority of chromatin with DNase I, cell lysates were subjected to affinity chromatography using a Nickel-Sepharose column (1.5 × 3 cm) for purifying zebrafish CSTB-like protein. Concentrated recombinant zebrafish CSTB-like protein was stored in phosphate buffer containing 10% glycerol at −80 °C.

### Cystatin B activity assay

The protease inhibitory property of recombinant zebrafish CSTB-like was examined based on the inhibition on papain activity for hydrolyzing azo-casein^42^. In brief, papain (1 mg/ml in 0.1 M phosphate buffer, pH7.4) was incubated with various amounts of recombinant zebrafish CSTB-like (concentrations ranging from 5 to 500 ng/μl) and incubated at 25 °C for 10 min before adding 125 µl of 0.5% azo-casein prepared in PBS. After incubating for another 30 min at 37 °C, reactions were stopped with 10% trichloroacetic acid and centrifuged. The supernatant was measured for absorbance at 440 nm. The percentage of remaining papain activity was calculated from the ratio of absorbance with and without zebrafish CSTB-like using the formula: 100 × (A_440nm_ with CSTB-like/A_440 nm_ without CSTB-like).

### *In vivo* injection of recombinant zebrafish CSTB-like

Recombinant zebrafish CSTB-like protein in PBS was mixed with Texa sRed-dextran and methylcellulose before being injected into the 56 hpf anesthetized FD larvae at the region surrounding swim bladder. Larvae injected with PBS served as injection control. Injected larvae were grown in embryo water and observed for swim bladder inflation at 5 dpf.

## Results

### Defective swim bladder was observed in FD larvae

**Figure 1:**
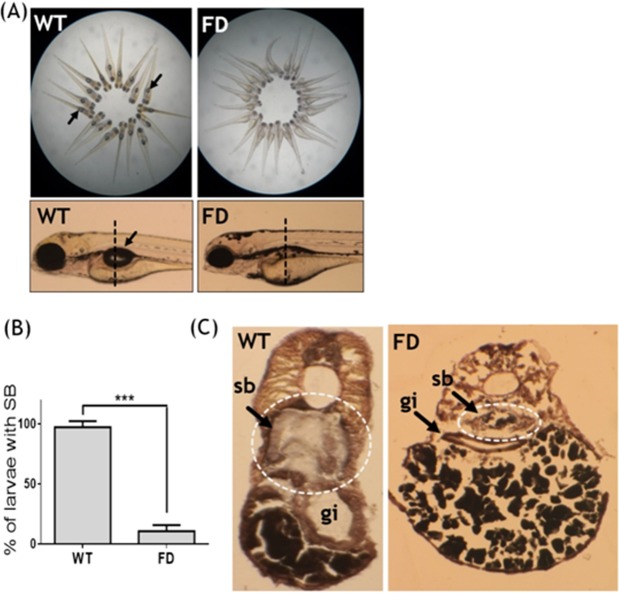
Defective swim bladder formation in FD larvae. Embryos of the zebrafish transgenic line Tg(*hsp*:EGFP-GGH) were heat-shocked at 9- and 24-hpf to induce folate deficiency and observed for swim bladder development at 5 dpf. (**A,B**) The clear bubble-like swim bladders (black arrows) were observed in almost all 5 dpf wild-type larvae but were absent in more than 80% of FD larvae. Data are reported as the means ± SD, n = 20 (20 independently conducted repeats with at least a total of 500 larvae for each group), ***p < 0.001. (**C**) The inflated chamber of swim bladder (the circled area indicated by arrows) is clearly seen in the elastin/collagen stained cross-sections (dash-line in (**A**)) prepared from wild-type control larvae, but not in FD larvae. WT, wild-type; FD, folate deficiency; sb, swim bladder; gi, gastrointestinal tract.

The previously established fluorescent transgenic line Tg(*hsp*:eGFP-GGH) will overexpress a fusion of eGFP with γ-glutamylhydrolase (GGH) upon heat-shock, which facilitates the exportation of intracellular folate and leads to folate deficiency with the extent positively correlated to its fluorescence intensity^17^. Normally, the inflated single-chambered swim bladder can be observed at 5 dpf in growing zebrafish larvae. However, more than 80% of FD larvae lacked a visible inflated swim bladder at 5 dpf (Fig. 1A,B). Cryo-sections prepared from 5-dpf wild-type larvae revealed an intact and properly inflated swim bladder (Fig. 1C). Contrarily, the swim bladder remnants resulting from unsuccessful inflation was observed in FD larvae. The absence of a successfully inflated swim bladder indicated impeded formation of swim bladder in FD larvae.

### The expression patterns of swim bladder tissue markers in early development were comparable between wild-type larvae and FD larvae

**Figure 2:**
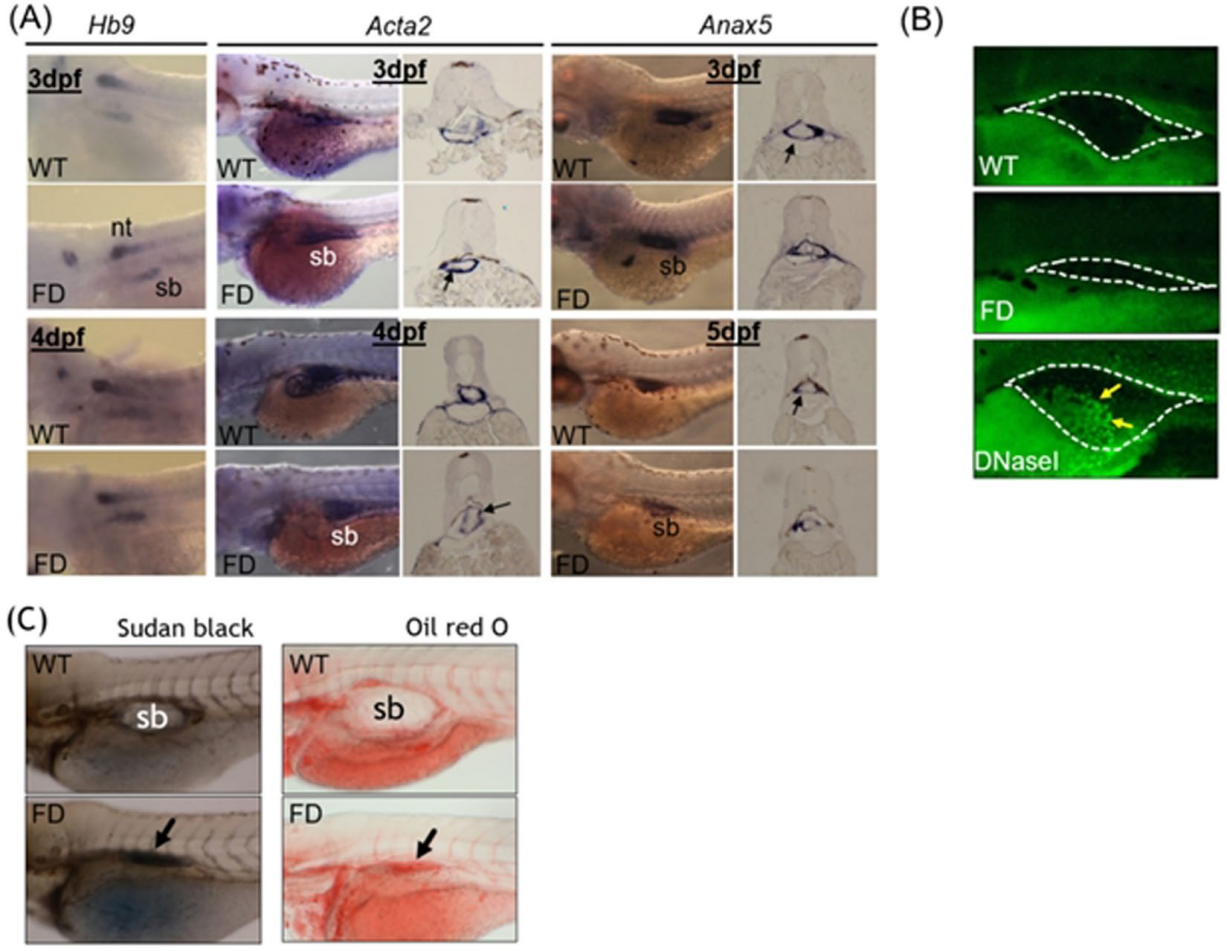
The tissue integrity and pathology of the swim bladder in wild-type and FD embryos and larvae. Heat-shocked larvae of both wild-type control and Tg(*hsp*:EGFP-GGH) were harvested at indicated stages post embryogenesis and analyzed for swim bladder development. (**A**) Embryos were subjected to WISH with tissues specific probes including *Hb9* (epithelial layer), *Acta2* (mesenchyme layer, smooth muscle) and *Anax5* (outer mesothelium layer). Cross-sections of the embryos after WISH staining revealed comparable intensity and morphology for the tissue layers composed of swim bladders (black arrows) in the early embryos. (**B**) Larvae at 5 dpf examined with TUNEL assay for apoptotic cells (arrows) revealed no positive signal in the swim bladder area (circled by dash-line) of both wild-type control and FD larvae. Embryos pre-treated with DNase I served as a positive control. (**C**) Larvae at 5 dpf were stained with Sudan black (left) and Oil red O (right) to reveal outlines of swim bladder (black arrows) in larvae. nt, neural tube; sb, swim bladder; WT, wild-type larvae with heat-shock; FD, FD larvae with heat-shock.

Whole-mount *in-situ* hybridization (WISH) is a strategy commonly used to examine the organ development and tissue integrity in developing embryos, in which the tissue morphology is visualized by the synthetic riboprobes complementary to the transcripts expressed specifically in the tissues of interest. Comparable expression patterns and intensity for all three probes (*Hb9*, *Acta2* and *Anax5*) corresponding to the three major tissue layers of zebrafish swim bladder were observed for both wild-type control and FD embryos at 3 dpf, nevertheless, the signals became weaker in FD larvae at later stages (Fig. 2A). Results from a TUNEL assay revealed no apparent signal for apoptosis in the swim bladder of both control and FD larvae (Fig. 2B). To investigate the integrity of swim bladder, larvae at 5 dpf were subjected to either Sudan black or Oil red O staining. With both staining methods, the swim bladder of the heat-shocked wild-type control larvae was revealed as a clear outline surrounding a morphologically sound and inflated air chamber area (Fig. 2C). On the contrary, a rather shrunk and darker stain patch was observed in the 5-dpf FD larvae, indicating a failed inflation of the air chamber possibly due to a swim bladder leakage. These data suggested that the development of swim bladder before 3 dpf was not affected by FD status. However, the tissue integrity of swim bladder might have been damaged in FD larvae.

### The transcripts of cathepsin L and a cystatin B-like (CSTB-like) protein were significantly increased and decreased, respectively, in FD larvae

**Figure 3:**
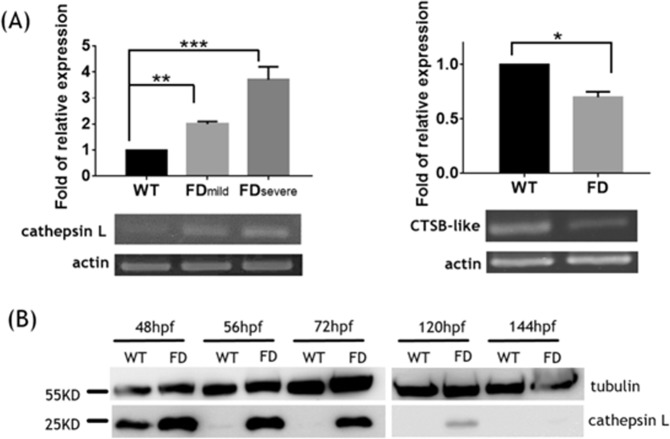
The altered expression of cathepsin L and CSTB-like in FD larvae. Both wild-type and FD larvae were collected at 5-dpf and analyzed for cathepsin L and cystatin B (CSTB)-like mRNA (**A**) and protein (**B**) expression levels. A dose-dependent increase in cathepsin L mRNA was observed in FD larvae when larvae were categorized into mild and severe groups based on the extent of folate deficiency (fluorescence intensity). The RT-PCR products for CSTB-like and the corresponding actin were run on two separate agarose gels. The larval protein extracts were analyzed on two separate Western blots. Full-length gels and blots are presented in Supplementary Figs S1–S3. WT, wild-type; FD, folate deficiency; Data are reported as the means ± SD, n ≥ 5. The statistical significance was calculated with Student’s t-test. *p < 0.05; **p < 0.01; ***p < 0.001.

To examine the impact of folate deficiency on the global gene expression profile during zebrafish embryogenesis, FD embryos at 14 hpf, 30 hpf and 120 hpf were subjected to microarray analysis. The expression profiles of both wild-type embryos and a transgenic line expressing a non-functional γ-glutamylhydrolase were also examined to serve as comparison^17^. Of all the genes displaying affected expression levels, cathepsin L and a CSTB-like protein (GenBank^TM^ accession number EH546362) were among those with the most apparent changes at 120 hpf (Table 1). The alteration in the mRNA levels of cathepsin L and zCSTB-like in FD larvae was confirmed with RT-PCR (Fig. 3A). Additionally, the cathepsin L transcript expression levels were increased and exhibited a positive correlation with the FD severity of the larvae. Western blotting results showed that cathepsin L was abundant in wild-type embryos at 48 hpf but vanished after 56 hpf. Contrarily, cathepsin L protein levels persisted in FD embryos until 120 hpf (Fig. 3B). The concurrent decrease of the zCSTB-like and increase of cathepsin L expression implied a possible elevation of cathepsin L activity in FD larvae.

**Table 1:** The fold of expressional change for cathepsin L and CSTB-like obtained from microarray data.

Access. No.	Description	Fold change in expression (FD/SDM)*		
		14 hpf	31 hpf	120 hpf
NM_131198	Danio rerio cathepsin L, 1b	−1.071	−1.119	+6.92
EH546362	Danio rerio cystatin B-like	−1.487	1.058	−107.157

^*^Reported is the averaged transcripts ratio between FD and SDM larvae of all probes (ranging from 1 to 5 probes for each gene) specific to the different sequence segments of the same gene. FD, larvae with folate deficiency; SDM, larvae expressing a non-functional GGH by site-directed mutation.

### The expression of cathepsin L and zCSTB-like was spatio-temporally specific in developing embryos

**Figure 4:**
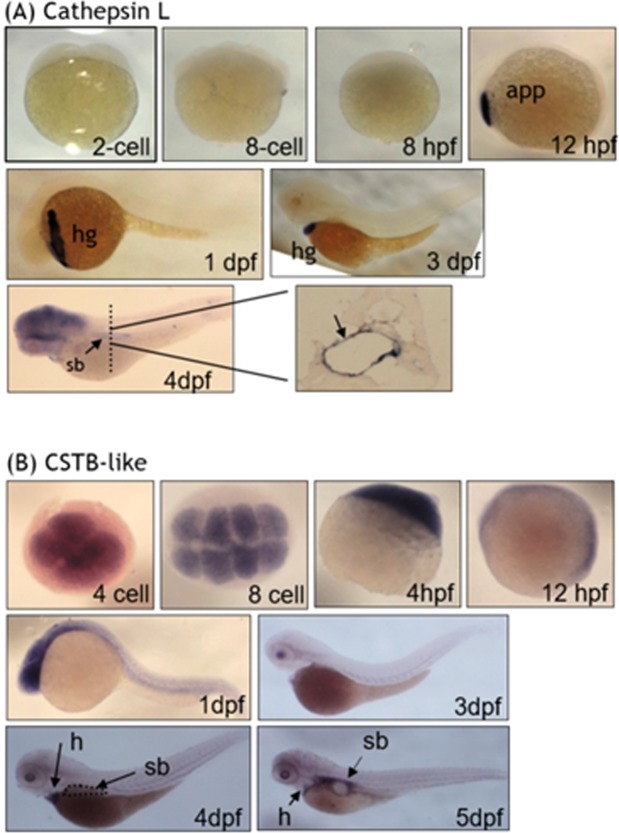
The expression of cathepsin L and CTSB-like during zebrafish embryogenesis. Wild-type embryos were analyzed with whole-mount *in situ* hybridization (WISH) at the indicated stages with antisense riboprobes against cathepsin L (**A**) and CSTB-like (**B**) for mRNA distribution. (**A**) No appreciable signal for cathepsin L was observed in embryos before 8 hpf. Signals were significant in 12 hpf embryos at anterior prechordal plate (app) and subsequently in hatching gland (hg) at later stages. Signals were also abundant in the head region and swim bladder (sb) of 4-dpf larvae, which can be observed more clearly in a post-WISH cross-section at the trunk region (dash line and black arrow in inset). (**B**) Intensive expression and homogenous distribution of CSTB-like transcripts were observed in embryos before 4 hpf. Signals became focused anteriorly in 1 dpf embryos and gradually diminished, as development proceeded, but nonetheless persistent in the trunk region along the vessels. Significant distribution appeared in the heart and the uninflated swim bladder area of 4 dpf larvae and became enriched in the region surrounding swim bladder at 5 dpf. app, anterior prechordal plate; h, heart; hg, hatching gland; sb, swim bladder.

The WISH with riboprobes specific to cathepsin L and CSTB-like revealed a stringent restriction for their expression in developing embryos. The WISH signal for cathepsin L was not detected in early embryos until 12 hpf (Fig. 4A). An intense signal patch appeared at the anterior prechordal plate, the most likely origin of the rostral cranial mesoderm and an integral part of the roof of the foregut. The signal was focused in the hatch gland of embryos at 1 to 3 dpf but disappeared later. At 4 dpf, the cathespin L signal was observed in head region and swim bladder. Contrarily, the CSTB-like transcripts were distributed homogenously in early embryos, but focused in the region surrounding swim bladder at later stages (Fig. 4B). Abundant and homogenous distribution of CSTB-like transcripts was observed in embryos before 4 hpf, even as early as the 4-cell stage, suggesting the deposition of maternal mRNA. Zebrafish CSTB-like signals became focused anteriorly in 1 dpf embryos and gradually diminished, as development proceeded. A signal of moderate intensity was observed in the trunk along the vessels at 3 dpf. Zebrafish CSTB-like transcripts were abundant in heart at 4 dpf but diminished at 5 dpf. Apparent signal was visible in the region surrounding the swim bladder at 4 dpf and became enriched at 5 dpf. The seemingly complementary expression patterns of cathepsin L and CSTB-like imply a potential mutual restraint in their functions between these two proteins during embryogenesis.

### The structure and activity of zebrafish CSTB-like are comparable to mammalian orthologs

**Figure 5:**
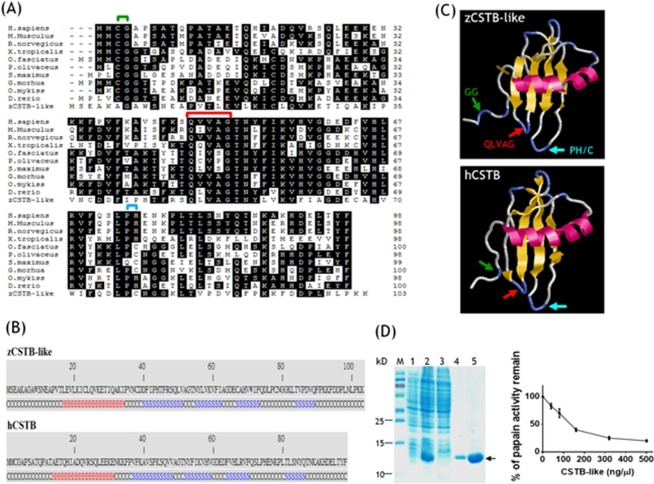
Structural and functional analysis of zebrafish CSTB-like protein. The coding sequence of zebrafish CSTB (zCSTB)-like was cloned, sequenced and compared with the cystatin B from other species. (**A**) Amino acid homology between zCSTB-like and cystatin B of other vertebrates, including zebrafish. The shaded letters indicate conserved amino acids among compared sequences. The conserved GG motif (green), PH/C motif (blue) and QxVxG (red) motifs are indicated by colored brackets. (**B**) The predicted secondary structures, based on the primary sequences of both zCSTB-like (upper) and human cystatin B (lower), are shown in colored letters. Black C, coil; red H, helix; blue S, strand. (**C**) The ribbon representation of the predicted 3D structures of both zebrafish CSTB-like (upper) and human cystatin B (lower). Both the secondary and 3D structures were predicted with on-line software server *I-tasser* (http://zhanglab.ccmb.med.umich.edu/I-TASSER/). The conserved motifs are indicated by colored arrows corresponding to the respective brackets indicated in (**A**). (**D**) Recombinant zebrafish CSTB-like was purified to at least 90% in purity, as judged from the SDS-PAGE result (lane 5, left) and analyzed for the inhibitory activity against papain (right). 1, total protein without IPTG induction; 2, total protein with IPTG induction; 3, the supernatant flow-through Ni-affinity column, 4, protein eluent from Ni-column; 5, purified CSTB-like (arrow); M, protein marker.

Amino acid sequence alignments reveal an approximate 45% homology and 32% identity between zebrafish CSTB-like and human cystatin B, which are the highest among the comparisons between zebrafish CSTB-like with other cystatins (Fig. 5A). Similarly, secondary structures, including a helix at the N-terminus followed by four consecutive β-strands, were predicted for both human cystatin B and zebrafish CSTB-like (Fig. 5B). The helix was situated right in front of the four-stranded β-sheet in the predicted 3D structure for both zCSTB-like and human CSTB (Fig. 5C). The structural motifs crucial for interacting with target enzymes, such as the Q-V-G motif, were also present in zebrafish CSTB-like^43^. The recombinant zebrafish CSTB-like was cloned, overexpressed in *E. coli* and purified to at least 90% purity (Fig. 5D). The purified recombinant zebrafish CSTB-like displayed inhibitory activity on the hydrolase activity of the cysteine protease papain. These results suggested structural and functional similarity between zebrafish CSTB-like and the proteins in the cystatin B family, supporting the cystatin B identity of zebrafish CSTB-like.

### Both the disturbed expression of cathepsin L/zCSTB-like and defective swim bladder were prevented by folate supplementation

**Figure 6:**
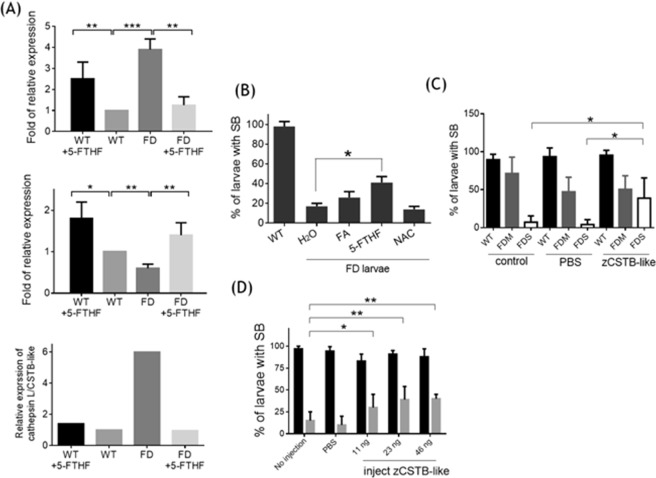
The rescuing effect of folate and recombinant zebrafish CSTB-like for FD-induced swim bladder anomaly and imbalanced expression of cathepsin L/CSTB-like. Zebrafish embryos were exposed to rescuing agents immediately after the first heat-shock at 9-hpf until 5 dpf before analysis. The embryo water containing folate or NAC was replenished every morning. (**A**) The cathepsin L and CSTB-like mRNA levels in embryos exposed to 1 mM 5-FTHF were analyzed with RT-PCR and quantified by comparing with the mRNA of actin. Data are presented as the ratio (fold of relative expression) to wild-type embryos. (**B**) Embryos exposed to FA, 5-FTHF and NAC were examined for the presence of intact larval swim bladder for rescuing effects. (**C**) Embryos with mild (FDM) or severe (FDS) folate deficiency were injected with 11 ng of recombinant zebrafish CSTB-like protein at 2 dpf and examined for the presence of inflated swim bladder at 5 dpf. (**D**) FDS embryos injected with the indicated doses of purified recombinant zebrafish CSTB-like protein were examined for the presence of inflated swim bladder. Data are reported as the means ± SD, n ≥ 4. The statistical significance was calculated with Student’s t-test. *p < 0.05; **p < 0.01; ***p < 0.001. 5-FTHF, 5-formyltetrahydrofolate; FA, folic acid; FD, folate-deficiency embryos; NAC, N-Acetyl cysteine; WT, heat-shocked wild type embryos.

Exposing FD larvae to 5-formyltetrahydrofolate (5-FTHF) right after inducing FD significantly decreased and increased the expression of cathepsin L and zCSTB-like, respectively (Fig. 6A). These alterations, in response to 5-FTHF rescue, had reversed the drastic increase in the ratio between the expression levels of cathepsin L and zCSTB-like in FD larvae (Fig. 6A, bottom panel). It should be noted that adding folate to wild-type embryos also increased the expression of both cathepsin L and zCSTB-like, yet the mRNA ratio between the two remains unchanged. The percentage of FD larvae with successfully inflated swim bladder was also increased when embryos were rescued with 5-FTHF (Fig. 6B). Unexpectedly, adding folic acid did not exert the same rescuing effect as with 5-FTHF. Adding N-acetylcysteine (NAC), a strong antioxidant, to embryo water did not prevent the impeded swim bladder formation induced by FD either (Fig. 6B). These results suggested that increased oxidative stress due to FD did not contribute to the defective swim bladder observed in FD larvae. These data supported the causal link between FD and the swim bladder anomaly, as well as the altered expression of cathepsin L and zCSTB-like observed in FD larvae. In addition, the expression of both cathepsin L and zCSTB-like was susceptible to folate modulation. Our results also revealed the diverse biological activities of different folate derivatives, at least between 5-FTHF and folic acid.

Injecting zCSTB-like protein prevented impaired swim bladder formation. The purified recombinant zCSTB-like protein was injected into 2-dpf larvae at the area adjacent to the expected swim bladder antecedent to examine the contribution of zCSTB-like in swim bladder formation. The percentage of FD larvae with successfully inflated swim bladder was significantly and dose-dependently increased with regard to the injected zCSTB-like concentration (Fig. 6C,D). These results supported the causal role of imbalanced cathepsin L/zCSTB-like activity for the impeded swim bladder formation in FD larvae.

## Discussion

Imbalanced protease/antiprotease activity is central to the current hypothesis for the pathomechanism of most respiratory tract diseases involving lung injury. In the current study, we showed cathepsin L and zCSTB-b complementarily express in developing zebrafish larvae at the antecedent of swim bladder and their expression profoundly modulate the integrity of swim bladder. Moreover, the increased cathepsin L and decreased zCSTB-b expression were observed in FD larvae. This imbalanced protease/antiprotease activity in response to FD contributed to the impaired swim bladder formation. This disturbed protease/antiprotease balance is also analogous to the pathomechanism of lung injury observed in emphysema, supporting the use of this model for related studies and drug discovery.

Proteases participate in tissue degradation and ECM remodeling in both normal and pathological conditions^8^. A balanced protease activity is mainly modulated by the proper expression of protease and antiprotease and is vital to maintaining ECM integrity^3,44^. Neutrophils are the major cells involved in COPD pathology and play a prominent role by releasing a multitude of mediators and proteases, such as elastase, which can orchestrate tissue destruction and chronic inflammation^45^. Studies also showed that alveolar macrophages, in response to chronic inflammation, increased cathepsin L synthesis and contributed to the destruction of ECM^3,46^. Cathepsin L is a lysosomal cysteine proteinase that hydrolyzes elastin and collagen, the two major components in ECM. Cathepsin L also proteolytically inactivates α1-anti-trypsin, a major regulator of human neutrophil elastase activity^2,47^. The zCSTB-like protein identified in the current report is structurally and functionally similar to cystatin B, a natural protein inhibitor of cathepsins^48,49^. Nevertheless, it should be noted that our WISH results with wild-type larvae revealed abundant cathepsin L transcripts in swim bladder and enriched zCSTB-like in the area surrounding swim bladder. In human, cystatin B is widely distributed among different cell types and tissues^50^. Albeit being considered to function intracellularly, cystatin B has also been found in extracellular fluid^51^. Our results support a role for this protease/antiprotease pair in zebrafish swim bladder formation. Further studies to identify the origin of the imbalanced cathepsin L/CSTB-like shall provide further insight to the pathomechanism underlying FD-induced defective swim bladder and hopefully COPD. We are fully aware that lack of sophisticated lung function measurements and the pathophenotypic discrepancy between the defective swim bladder and emphysema are the limitations for this FD zebrafish model. Nevertheless, considering the fact that inflation of the swim bladder requires the airflow along with its retention inside the chamber, the integral appearance of the air-filled swim bladder in transparent larvae is a direct and faithful reflection of air sac function and ECM integrity. Thus, it can provide a convenient measurement and assessment on the efficacy of the interventions under test. This model could be an efficient *in vivo* platform for drug discovery on COPD if the results are extrapolated cautiously.

Our data also substantiate and support for the connection between an individual’s folate status and pulmonary wellness. That 5-FTHF supplementation significantly prevented FD-induced swim bladder anomaly and altered expression of cathepsin L and zCSTB-like unveils a possible path for folate to modulate ECM integrity, which may be differentially reflected in the pulmonary function between physiological and pathological conditions. Although FD has not been considered a direct risk factor for COPD, the influence of maternal folate intake on fetal pulmonary integrity and pulmonary diseases has been documented^52^. Previous studies also showed that the expression of proteases, specifically metalloproteases and cathepsins, in peripheral tissues were increased in rheumatoid arthritis patients receiving methotrexate^53^. Methotrexate is a powerful dihydrofolate reductase (DHFR) inhibitor. DHFR converts folic acid and dihydrofolate to tetrahydrofolate, the active coenzyme. 5-FTHF is the reduced folate derivative often used in methotrexate combined therapy for treating cancers. Therefore, it is surmisable that FD increases an individual’s vulnerability to environmental toxins and/or other risk factors causing COPD. This speculation is supported by the increased mortality and the rate of malformation when FD embryos were exposed to caffeine and ethanol as compared to wild-type control (unpublished data). One other interesting observation is that both cathepsin L and zCSTB-like transcripts were increased in wild-type larvae exposed to 5-FTHF but the ratio between this protease/antiprotease pair remained unaffected. These results help further emphasize the importance of maintaining protease/antiprotease balance in order to sustain tissue integrity. Understanding how the expressions of these protease/antiprotease are regulated, and how folate functions in helping maintain ECM integrity and lung health shall be beneficial to optimizing disease management and improving healthcare regimens for COPD patients.

While adding 5-FTHF significantly prevented the FD-induced swim bladder anomaly, supplementing with folic acid failed to rescue was rather unexpected. Folic acid is the inactive and oxidized form of folate commonly included in the food and nutrient supplement available in the neighborhood drug stores. As aforementioned, folic acid needs to be further reduced by DHFR in order to be physiologically active. Unlike reduced folate, folic acid lacks appreciable antioxidant activity, raising the possibility that the defective swim bladder formation was due to increased oxidative stress caused by FD^17^. Nevertheless, the presence of antioxidant NAC did not prevent against swim bladder impairment observed in FD larvae in our current studies. These results indicated that the swim bladder anomaly was possibly not caused by the direct elevated oxidative stress in FD larvae. The other clue comes from the altered expression of cathepsin L and CSTB-like observed in FD larvae, which implies a disturbed intracellular gene activity. Gene activity is known to be modulated by intracellular methylation potential (*i.e*. the levels of S-adenosylmethionine and 5-methyltetrahydrofolate) via epigenetic control. We had previously shown that zebrafish DHFR is structurally and functionally comparable to mammalian orthologs and is abundant in developing embryos^54,55^. Therefore, the supplemented folic acid should have been converted to reduced folate and utilized by larvae. However, it should be noted that folic acid is a less effective substrate of DHFR. The presence of high-dose of folic acid had been shown to repress the conversion of dihydrofolate to tetrahydrofolate *in vitro*^54^. Moreover, elevated cellular levels of dihydrofolate had been suggested to cause inhibition on the activity of methylenetetrahydrofolate reductase, the enzyme catalyzing the production of 5-methyltetrahydrofolate^56^. Therefore, the disturbed methylation potential caused by FD and folic acid supplementation might have contributed to the impaired expression of cathepsin L/cystatin B expression, as well as the unsuccessful rescues with folic acid, respectively. Previously, we had shown that supplementing with different folate derivatives exerted different physiological impacts on the receiving organisms, suggesting distinct and non-interchangeable activity between different folate derivatives^55,57^. The notion that folic acid failed to rescue FD-induced swim bladder malformation further supports the diverse activities among different folate derivatives. Further investigation, especially characterization on the distribution of different one-carbon derivatives in folate pool under the circumstance of FD and folate supplementation shall provide additional insights to the pathological mechanisms underlying FD and for the best and safe use of folate supplements.

In conclusion, we showed that the concurrent increase and decrease of cathepsin L and CSTB-like expression, respectively, contributed to the impeded swim bladder formation observed in FD larvae. Local injection of recombinant zebrafish CSTB-like protein, which is structurally and functionally comparable to human cystatin B, successfully prevented the FD-induced swim bladder malformation. Diverse effectiveness of rescue mediated by different folate adducts were also observed, in which successful rescue was found when 5-FTHF supplementation, but not folic acid, was used. Our studies provide the evidence to surmise the connection between the individual’s folate status to ECM integrity, and potentially pulmonary function, by intervening protease/antiprotease balance.

## Supplementary information

Supplementary Figures S1–S3
