# Supplementary material for: A novel zebrafish model to emulate lung injury by folate deficiency-induced swim bladder defectiveness and protease/antiprotease expression imbalance
Source: Sci Rep. 2019 Sep 2;9:12633. doi: 10.1038/s41598-019-49152-7 (PMC6718381; doi:10.1038/s41598-019-49152-7)
Supplement: Supplementary file 1 — Supplementary Figures S1–S3 [file 41598_2019_49152_MOESM1_ESM.pdf]

A novel zebrafish model to emulate lung injury by folate deficiency-induced swim bladder defectiveness and protease/antiprotease expression imbalance

Gang-Hui Lee[1,2#], Nai-Wei Cheng[1#], Hsin-Hsuan Yu[1], Jen-Ning Tsai[3,4#], Tsunglin Liu[5], Zhi-Hong Wen[6], Bing-Hung Chen[7,8,9,10,*], and Tzu-Fun Fu[1,2,*]

[1]Department of Medical Laboratory Science and Biotechnology, National Cheng Kung University, Tainan, Taiwan; [2]Institute of Basic Medical Science, National Cheng Kung University, Tainan, Taiwan; [3]Department of Medical Laboratory and Biotechnology and [4]Clinical Laboratory, Chung Shan Medical University Hospital, Chung Shan Medical University, Taichung, Taiwan; [5]Institute of Bioinformatics and Biosignal Transduction, National Cheng Kung University, Tainan, Taiwan; [6]Department of Marine Biotechnology and Resources, Asia-Pacific Ocean Research Center, National Sun Yat-sen University, Kaohsiung, Taiwan; [7]Department of Biotechnology, Kaohsiung Medical University, Kaohsiung, Taiwan; [8]Department of Medical Research, Kaohsiung Medical University Hospital, Kaohsiung Medical University, Kaohsiung, Taiwan; [9]Centers for Biomarkers and Biotech Drugs, Kaohsiung Medical University, Kaohsiung, Taiwan; [10]The Institute of Biomedical Sciences, National Sun Yat-sen University, Kaohsiung, Taiwan

* To whom correspondence should be addressed: Tzu-Fun Fu: Department of Medical Laboratory Science and Biotechnology, National Cheng Kung University, College of Medicine, No.1, University Road, Tainan 701, Taiwan. Tel.: 886-6-2353535 (ext. 5795); Fax: 886-6-236-3956; E-mail: tffu@mail.ncku.edu.tw; Bing-Hung Chen: Department of Biotechnology, Kaohsiung Medical University, Kaohsiung, 800, Taiwan. Tel: 886-7-3121101 (ext. 2676); E-mail: bhchen@kmu.edu.tw

[#] These authors contribute equally to this work.

(Supplementary Figures)

Figure S1

(A)

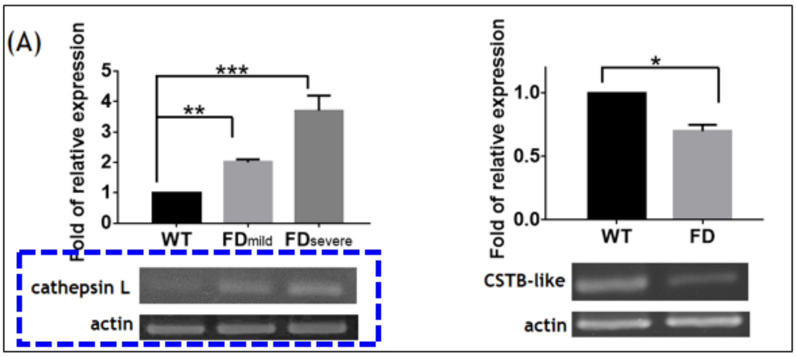

(B)

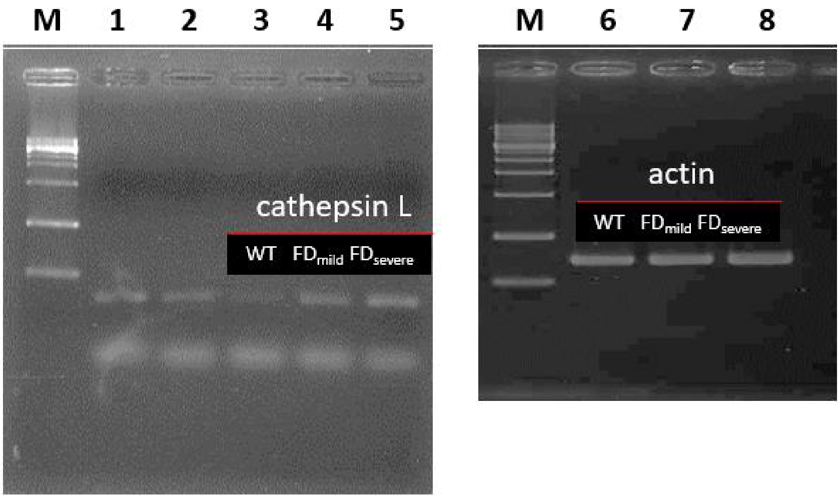

Figure S1. The original full-length RT-PCR gel results for cathepsin L/actin analysis. (A) The original cropped gel results (blue dotted rectangle) as shown in Figure 3A in the manuscript. (B) The original full-length gels for the analyses on cathepsin L (left) and actin (right). M, marker.

Figure S2

(A)

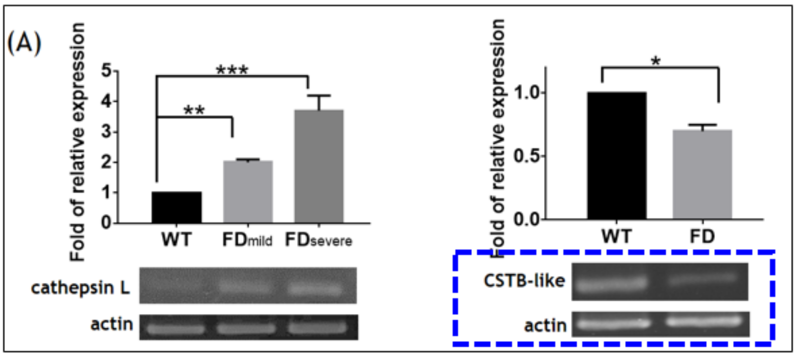

(B)

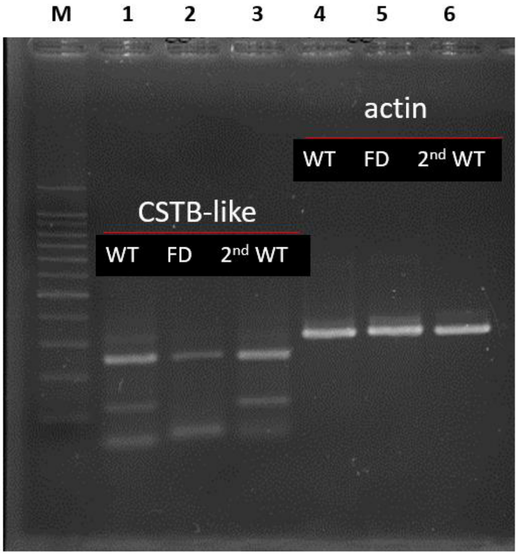

Figure S2. The original full-length RT-PCR gel result for CSTB-like/actin analysis. (A) The original cropped gel results (blue dotted rectangle) as shown in Figure 3A in the manuscript. (B) The original full-length gel for the analyses on CSTB-like (left) and actin (right). M, marker.

Figure S3

(A)

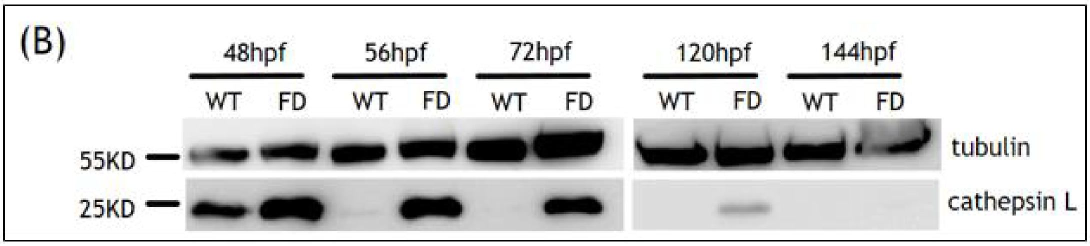

(B)

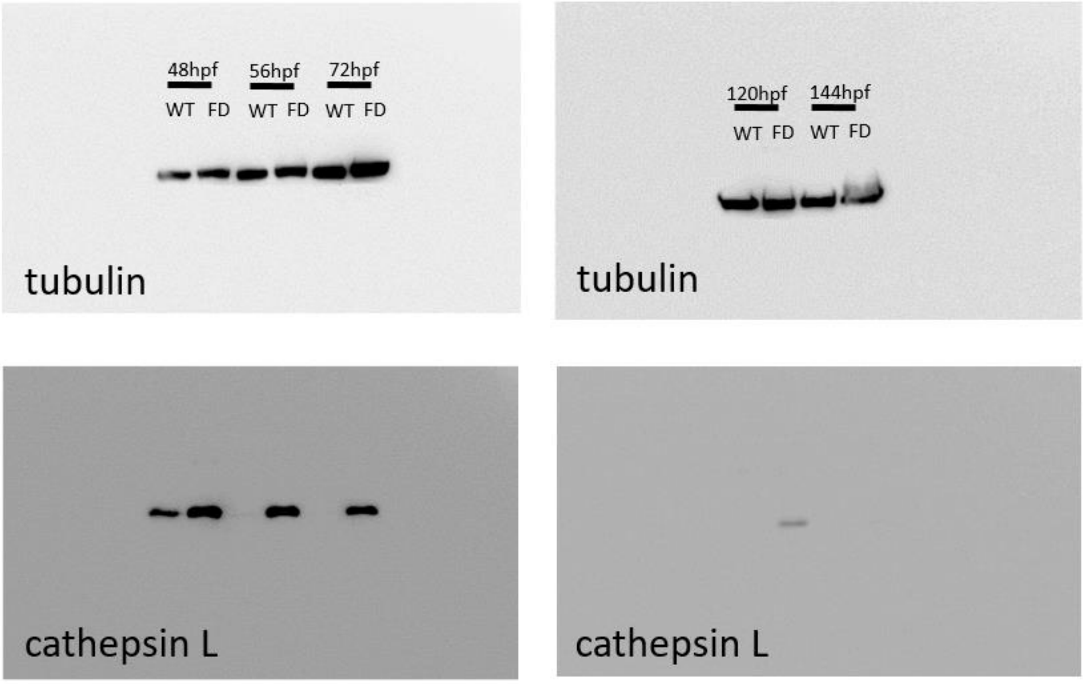

Figure S3. The original full-length RT-PCR gel results for CSTB-like/actin analysis. (A) The original cropped Western blotting results as shown in Figure 3B in the manuscript. (B) The original full-length Western blots for the analyses on tubulin (top blots) and cathepsin L (bottom blots).
